# Frequency of *Chlamydia trachomatis* infection in cervical intraepithelial lesions and the status of cytological p16/Ki-67 dual-staining

**DOI:** 10.1186/s13027-016-0111-8

**Published:** 2017-01-06

**Authors:** R. Robial, A. Longatto-Filho, C. M. Roteli-Martins, M. F. Silveira, D. Stauffert, G. G. Ribeiro, I. M. Linhares, M. Tacla, M. A. Zonta, E. C. Baracat

**Affiliations:** 1 Division of Gynecologic Clinic, Faculdade de Medicina da Universidade de São Paulo, São Paulo, Brazil; 2 Laboratory of Medical Investigation (LIM) 14, Faculty of Medicine, University of São Paulo, São Paulo, Brazil; 3 Molecular Oncology Research Center, Barretos Cancer Hospital, Pio XII Foundation, Barretos, Brazil; 4 Life and Health Sciences Research Institute (ICVS), School of Health Sciences, University of Minho, Braga, Portugal; 5 ICVS/3B’s - PT Government Associate Laboratory, Braga/Guimarães, Portugal; 6 Leonor M De Barros Hospital – Secretaria de Saúde de São Paulo, São Paulo, Brazil; 7 Federal University of Pelotas – Pelotas, Rio Grande do Sul, Brazil; 8 School of Pharmaceutical Sciences of Ribeirão Preto University of São Paulo, Ribeirão Preto, Brazil; 9 Discipline of Infectology, Federal University of São Paulo, São Paulo, Brazil

**Keywords:** *Chlamydia*, Cervical cancer, Immunocytochemistry, ki 67

## Abstract

**Background:**

*Chlamydia trachomatis* (Ct) is not a disease subject to mandatory reporting in Brazil, and the prevalence rate of this genital infection varies according to the region in which studies are conducted, as well as by the detection technique employed. Ct has been associated with persistence of Human papillomavirus (HPV) infection and the facilitation of cervical carcinoma development. We evaluated the *Chlamydia trachomatis* infection and its association with cytology, p16/Ki-67 dual-stained cytology and cervical intraepithelial lesions status in a screening cohort in Brazil.

**Methods:**

This was a cross-sectional study of 1481 cervical samples from asymptomatic women aged 18 to 64. Samples were collected for liquid-based cytology and Ct detection by polymerase chain reaction. p16/Ki-67 double staining was performed on samples with abnormal cytology. Statistical analysis was by chi-square and likelihood-ratio tests. Odds ratio (OR) and 95% confidence intervals (95% CI) were determined.

**Results:**

The frequency of Ct was 15.6% and its presence was not associated with detection of p16/Ki-67 [OR = 1.35 (0.5–3.4)]. There was also no association between abnormal cervical cytology and *Ct*-positivity [OR = 1.21 (0.46–3.2)]. Associations were observed between p16/Ki-67 and high-grade lesions detected by cytology and in biopsies [OR = 3.55 (1.50–8.42) and OR = 19.00 (0.6–7.2), respectively].

**Conclusions:**

The asymptomatic women in our study had a high frequency of Ct infection but this was not associated with p16/Ki-67 detection in samples with abnormal cytology. The expression of p16/Ki-67 was highest in women with high-grade CIN (*p* = 0.003).

## Background

*Chlamydia trachomatis* (Ct) is not a disease subject to mandatory reporting in Brazil, and the prevalence rate of this genital infection varies according to the region in which studies are conducted, as well as by the detection technique employed [1–4]. Ct has been associated with persistence of Human papillomavirus (HPV) infection and the facilitation of cervical carcinoma development. It has also been hypothesized that Ct may promote HPV access and entry in to the basal layer of the epithelium by inducing chronic inflammation, cervical hypertrophy and metaplasia. Metaplasia is a preferred HPV target [5]. and these biological effects increase the risk of cervical cell transformation and the persistence of infection by oncogenic HPV types. A concomitant Ct and HPV infection is hypothesized to increase the expression of Ki-67 (a marker for proliferation of the cervical epithelium) by HPV-generated mitogenic actions and by the anti-apoptotic activity elicited by both infectious agents [5]. Ct may also increase protein expression of oncogenic HPV16 in low-grade lesions [5].

Cervical cancer screening via cytology may reduce mortality by up to 50% under appropriate conditions and when solid population coverage is implemented; however, the sensitivity of cytology to detect CIN or invasive cancer may be <60% [6].

An increased understanding of how HPV contributes to cervical cell neoplastic transformation led to the routine use of biomarkers, such as p16, to identify HPV-mediated transformation. Studies have suggested that analysis for the p16 biomarker by cytology and histology in cervical lesions may have clinical utility by optimizing the efficiency of cytological tests [7]. Such findings have ensured greater efficiency in the early detection of cervical cancer. However, since some normal cervical cells may express p16, it is necessary to conduct additional morphologic evaluations. A recently developed p16 and Ki-67 detection kit enables the recognition of abnormal cells based on the double staining of both markers in the same cell, thereby possibly reducing the need for morphologic interpretation [7].

Recent publications claims that p16/Ki-67 detect oncogenic molecular changes induced by a persistent HPV infection in the cell through detection of concordant expression of the tumors suppressor protein p16 (also cyclin-dependent kinase inhibitor 2A) and the proliferation marker Ki-67 in the same cell, which are mutually exclusive in cells with a normal cell cycle. Since the latter cells are consistently cell cycle arrested, the combination of antibodies detecting p16INK4a and the cell cycle progression marker Ki-67 in one cell allows for the unequivocal identification of truly HPV-transformed cervical cells [8, 9].

The present study was conducted to analyze *Chlamydia trachomatis* infection and its status with cytology, p16/Ki-67 dual-stained cytology and CIN status in a screening cohort in Brazil.

## Methods

**Fig. 1:**
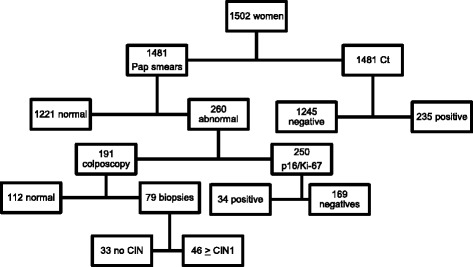
Description of Biopsies According to Presence of *Chlamydia trachomatis*. Flowchart for the tests performed. *CIN* cervical intraepithelial neoplasia, *Ct Chlamydia trachomatis*

This was a cross-sectional study of 1481 cervical samples preserved in liquid media, collected from asymptomatic women aged 18 to 64 and enrolled in a cervical cancer screening project in one clinical center (Leonor Mendes de Barros Hospital) in Sao Paulo, Brazil. Pregnant or hysterectomized women or women being treated for cervical neoplasia were excluded from the study. All women included in the study filled out a questionnaire on risk factors for cervical cancer. Cervical samples were collected at the ectocervix/endocervical junction with a Rover’s Cervex-Brush and the material was placed in the liquid preservative medium (Fig. 1).

### Cytology slides preparation

The entire brush head was introduced into the tube containing the BD SurePath™ liquid (BD Diagnostics – TriPath, Burlington, NC, USA), and sent to laboratory. The slides were prepared according to the manufacturer’s instructions, using *BD PrepMate*™ and *BD PrepStain*™. All liquid-based preparations were performed with a semi-automated methodology, according to the manufacturer’s instructions and manually analyzed.

Cytological changes were classified according to the 2014 Bethesda System [10] A colposcopy examination was requested for women with cytological abnormalities equal to or worse than Atypical Squamous Cells of Undetermined Significance (ASCUS).

### *Chlamydia trachomatis* detection

One thousand four hundred eighty one samples were collected for Ct detection in the BD cytology residual medium with the *BD ProbeTec* Amplified DNA Test, according to the manufacturer’s instructions.

### Immunocytochemistry of p16/Ki-67 dual-staining

The CINtec PLUS p16/Ki-67 dual-staining analysis was conducted in cytology results equal to or worse than ASCUS.

Immunostaining was performed according to the manufacturer’s instructions (Ventana, currently *Roche Diagnostics GmbH*, Mannheim, Germany).

The slides were analyzed searching for the presence of cervical epithelial cytoplasmic brown color and a red nuclear color, indicating concurrent expression of p16 and Ki-67. The presence of one or more cervical epithelial cells with both brown and red color was interpreted as a positive test result. The analyses were made by one of the authors (ALF).

### Statistics

Sample size for this study was based on the study of Santos et al. (2002), which detected a Ct prevalence of 20.7% in women aged 14 to 63. To obtain a similar frequency, with 95% CI and 2% error, the present study would have to include 1576 women. However, due to reading failures and material losses, 1481 women were analyzed, thereby attributing an error of 2.07% to the study.

Statistical analysis was conducted based on chi-squared or likelihood-ratio tests. Odds ratio (OR) and the respective 95% confidence intervals (95% CI) were estimated.

## Results

### Characteristics of the study population

The ages of the women enrolled for cervical cancer screening ranged from 18 to 64 years, with a mean of 40 years (SD = 11.2). Most women self-identified as White (58%), 33% were Afro-American while the remaining 9% were mixed race. The majority of subjects (57%) had a stable partner. The mean age at first sexual intercourse was 18 years (SD = 3.9). The number of lifetime sexual partners ranged from 1 to 30, with a mean of 3 partners (SD = 2.5).

Among the 1,481 women, 772 reported to have had at least one prior Pap test examination. Among of the 1351 women who answered the question regarding previous sexually transmitted disease (STD), 87% reported not to have had any prior STD. In relation to smoking, 64% of the women reported to never have smoked, and 17% of the women stopped smoking in the past 12 months.

### Frequency of Ct and association with p16/Ki-67

The Ct frequency was of 15.9%. No association was found between Ct and p16/Ki-67 positivity in women with abnormal cytology (Table 1). Only age (Table 2) and the number of lifetime sexual partners (Table 3) showed an association with detection of Ct (Tables 1 and 2).

**Table 1:** Association of *Chlamydia trachomatis* with p16/Ki-67

Variable	Chlamydia				OR (95% CI)	Total	*P*
	Negative		Positive				
	*n*	%	*n*	%			
p16/Ki67							
Negative	140	84.3	26	15.7	1	166	0.529
Positive	28	80.0	7	20.0	1.35 (0.5–3.4)	35

**Table 2:** Frequency of *Chlamydia trachomatis* according to demographic variables

Variable	*Chlamydia trachomatis*				OR (95% CI)	Total	*p*
	Negative		Positive				
	*n*	%	*n*	%			
Age Group							
≤ 25	125	88.7	16	11.3	1	141	0.040
26–35	295	84.5	54	15.5	1.43 (0.8–2.6)	349	
36–45	352	80.2	87	19.8	1.93 (1.1–3.4)	439	
46–55	352	86.3	56	13.7	1.24 (0.7–2.2)	408	
> 55	126	87.5	18	12.7	1.12 (0.5–2.3)	144	
Schooling (Years)							
< 8	392	82.9	81	17.1	1	473	0.156
8–11	634	86.4	100	13.6	0.76 (0.6–1.0)	734	
> 11	182	82.4	39	17.6	1.04 (0.7–1.6)	221	
Marital Status							
Single	530	85.8	88	14.2	1	618	0.211
Living with partner	696	83.4	139	16.6	1.20 (0.9–1.6)	835	
Smoking							
Never	793	85.7	132	14.3	1	925	0.128
Currently	234	81.0	55	19.0	1.41 (1.0–2.0)	289	
Previously	202	83.1	41	16.9	1.22 (0.8–1.8)	243

**Table 3:** Frequency of *Chlamydia trachomatis* according to sexual and obstetric history

Age at sexual debut (years)							
< 15	299	84.0	57	16.0	1	356	0.875
16–19	622	84.6	113	15.4	0.95 (0.7–1.3)	735	
> 19	298	85.4	51	14.6	0.90 (0.6–1.3)	349	
Number of Lifetime Partners							
1–4	1022	85.3	176	14.7	1	1198	0.015
5–10	195	82.3	42	17.7	1.25 (0.9–1.8)	237	
> 10	12	60.0	8	40.0	3.87 (1.56–9.61)	20	
Number of Partners in the Past Year							
0–1	1160	84.7	209	15.3	1	1369	0.709
2–4	75	81.5	17	18.5	1.26 (0.7–2.2)	92	
> 4	5	83.3	1	16.7	1.11 (0.1–9.5)	6	
Parity							
0	230	85.2	40	14.8	1	270	0.804
1–2	543	85.0	96	15.0	1.02 (0.7–1.5)	639	
> 2	443	83.7	86	16.3	1.12 (0.7–1.7)	529	
Prior STD							
No	999	85.5	170	14.5	1	1169	0.083
Yes	130	80.2	32	19.8	1.45 (0.9–2.2)	162	

In particular women between 35 and 45 years old had a higher rate of Ct positivity when compared to women in other age groups. Women with over 10 lifetime partners had the highest rate of being positive for Ct (*p* = 0.015 OR = 4.14) (Tables 3, 4 and 5).

**Table 4:** Result of the multiple logistic regression model to explain chlamydia positivity

Variable	OR	IC (95%)	*p*
Age group (years)			
≤ 25	1.00		
> 25 e ≤ 35	1.50	0.81–2.77	0.195
> 35 e ≤ 45	2.01	1.12–3.63	0.020
> 45 e ≤ 55	1.35	0.73–2.47	0.339
> 55	1.26	0.61–2.63	0.532
Number of partners in life			
1–4	1.00		
5–10	1.20	0.83–1.75	0.332
> 10	4.14	1.66–10.36	0.002

**Table 5:** Association between Pap smears and *Chlamydia trachomatis* infection

Variable	*Chlamydia trachomatis*				OR (95% CI)	Total	*P*
	Negative		Positive				
	*n*	%	*n*	%			
Cytology							
Normal/Inflammatory	1038	84.8%	186	15.2	1	1224	0.935
ASCUS	45	80.4	11	19.6	1.36 (0.7–2.7)	56	
LSIL	134	83.8	26	16.2	1.08 (0.7–1.7)	160	
HSIL	23	82.1	5	17.9	1.21 (0.46–3.2)	28	
AGC	6	85.7	1	14.3	0.93 (0.1–7.8)	7	
SCC	1	100.0	0	0	#		

Among the histological results, women diagnosed with CIN1 had a higher frequency of Ct compared to women with other cytological findings, but the difference was not statistically significant (*p* = 0.112) (Table 6).

**Table 6:** Association between biopsies and *Chlamydia trachomatis* infection

Variable	*Chlamydia trachomatis*				OR (95% CI)	Total	*P*
	Negative		Positive				
	*n*	%	*n*	%			
Histology							
Normal	86	79.6%	22	20.4	1	108	0.194
Cervicitis	30	93.8	2	6.2	0.26 (0.06–1.18)	32	
CIN 1	21	77.8	6	22.2	1.12 (0.40–3.1)	27	
CIN2/3	10	76.9	3	23.1	1.17 (0.3–4.63)	13	

*CIN 1* cervical intraepithelial neoplasia 1

*CIN2/3* cervical intraepithelial neoplasia 2 and 3

Regarding the cytological findings, there was an association between p16/Ki-67 positivity and detection of high-grade lesions (OR = 3.55), confirmed in women with CIN 2/3 who were highly positive for p16/Ki67 (Tables 7 and 8).

**Table 7:** Association of p16/Ki-67 expression with Low-grade and High-Grade Cytological Diagnosis

Variable	Cytology				OR (95% CI)	Total	*P*
	ASCUS/LSIL		HSIL				
	*n*	%	*n*	%			
P16/ki67							
Negative	141	88.1	19	11.9	1.00	160	0.003
Positive	23	67.6	11	32.4	3.55 (1.50–8.42)	34

**Table 8:** Association of p16/Ki67 Expression with colposcopy and histological results

Variable	P16/Ki67				OR (95% CI)	Total	*P*
	Negative		Positive				
	*n*	%	*n*	%			
Colposcopy/Biopsies							
Normal Colposcopy/No Biopsy	76	90.5	8	9.5	1.00	84	<0.001
Cervicitis	22	81.5	5	18.5	2.16 (0.64–7.27)	27	
CIN 1	20	76.9	6	23.1	2.85 (0.89–9.16)	26	
CIN 2/3	4	33.3	8	66.7	19.00 (4.67–77.36)	12	
Total	122	81.9	27	18.1		149	

Chi-squared test; OR and IC (95%) calculated using bivariate logistic regression

*CIN1* cervical intraepithelial neoplasia 1

*CIN2/3* cervical intraepithelial neoplasia 2 and 3

## Discussion

Prior studies have investigated the role of microorganisms like Ct that cause a chronic inflammation as a potential risk factor in the transmission and persistence of HPV, as well as contributing to the progression of cervical carcinogenesis [11, 12]. Our study population had a 15.6% frequency of Ct infection, as assessed by analysis of the liquid preservative media utilized for cervical cancer screening. However, we did not detect an association between p16/Ki-67 expression and Ct in women with CIN.

In Brazil, four studies conducted in different states, one in Goiás, two in Paraná and one in Rio Grande do Norte showed a Ct prevalence of 10.9, 10.7, 12.7 and 10.9%, respectively, also compatible with results in the present study. A study conducted in the city of Manaus had a higher prevalence of 20.7%, and may be explained by regional or socioeconomic reasons [4, 13–15].

Ct is not an infection subjected to mandatory reporting in Brazil. Additionally, there are no specific programs implemented by the Ministry of Health to encourage screening for this infection. Because it is asymptomatic in most women, it is likely to be under-diagnosed. As such, its true prevalence in this country remains ambiguous. Asymptomatic untested women are obviously not treated. This may account for the high number of positive women in our study. The Ct prevalence estimates must be interpreted within the context of national and cultural differences, of sexual behavior and the health system. Brazil is a very large country whose population has different cultural and socioeconomic development levels, which most likely explains the reported regional differences [16–18].

Age was the only demographic variable in this study that was associated with Ct infection. Women aged 35 to 45 years had the highest likelihood of being Ct positive. This observation differs from studies conducted in the United States, where the highest prevalence was among women aged 15 to 24 years. In the United States screening programs for Ct are available for women aged 25 years or younger, and for women at risk for this infection. Increased testing and treatment may explain the overall lower prevalence, as well as the lower rate found among women aged 25 years and older in the United States as compared to Brazil [12].

When assessing other demographic factors, such as years of schooling, marital status and smoking, there was no positive association with Ct presence, similar to what has been reported in other national studies [13].

The analysis of sexual and obstetric history reveals that only the number of lifetime partners was associated with being positive for Ct. Women with more than 10 partners had a four fold higher rate of positivity than did women with 1–4 partners. This is explained by the fact that Ct is a sexually transmitted disease, and infection is associated with high-risk sexual behavior. This association was also demonstrated in a prior Brazilian study [13].

Because it is an infection in which 90% of infected women are asymptomatic but, nevertheless, can have serious consequences for both reproductive and fetal health, Ct has been considered the non-viral STD with the highest burden for public health. Several lines of evidence suggest that screening for Ct is cost effective when its prevalence is above 3%. The few Brazilian studies conducted until now clearly justify the implementation of systematic screening of women for this infection [18].

We did not observe an association between abnormal cervical cytology and the presence of Ct. An analysis of women with abnormal cytological results revealed a higher prevalence of Ct infection in women with atypical lesions (ASCUS), followed by low-grade lesions (LSIL), in which almost 20% were Ct positive. These observed rates were similar to other Brazilian studies with women in the same age group [14].

In women who underwent a colposcopy examination a greater prevalence of Ct positivity was found in women with CIN1. However, the lack of statistical significance may be due to the low number of biopsies conducted after the cytology results. No prior Brazilian studies have assessed this association.

Our study was limited by the lack of HPV testing, due to the limited amount of residual material available. It is necessary, however, to take into consideration the “status” of HPV infection and the natural history of cervical cancer when determining the role of Ct as a co-factor in CIN or invasive cancer. HPV infection is a highly prevalent sexually transmitted viral infection, whereas Ct is the most commonly sexually transmitted bacterial infection, and co-infections with both microorganisms are quite common. No association was found in this study between Ct infection and cytology and positive biopsies for CIN, presumed positive for HPV. These results differ from other studies that found a positive association between HPV and Ct detected by serology [19, 20],. However it was similar to other studies using the same methodology that did not find any association between *Ct* DNA detection and subsequent risk of CIN2/3 [21, 22].

This study utilized p16/Ki-67 expression as a marker of transforming HPV infections. The study was designed to explore the possible association between a *Ct* infection and consequences of a persistent HPV infection by the use of p16/Ki-67, instead of testing for HPV DNA. In most cases, HPV DNA is unable to distinguish between persistent and transient infection. We conclude that there is no association between Ct and p16/Ki-67 positivity in women with abnormal cytology [22].

Most women with cytological findings were negative for p16/Ki-67 expression. However, approximately one fourth of the abnormal cytological results were positive for p16/Ki-67 double staining, a finding compatible with publications demonstrating that women with p16/Ki-67 positivity were also the women infected with HPV. Women with HSIL had a higher positivity prevalence (37%) than did women with LSIL. p16/Ki-67 positivity is therefore associated with a threefold higher prevalence in women with a high-grade lesion. These results were comparable to a recent study assessing the use of p16/Ki-67 in a sample from women referred for colposcopy and finding that this marker indeed had a higher positivity in women with high-grade lesions [23, 24].

Evaluation of the association of p16/Ki-67 expression with histopathological results also revealed a higher rate of p16/Ki-67 positivity in women with CIN2 or more advanced lesions. p16/Ki-67 positivity increased with the seriousness of the lesion. When the biopsy result revealed chronic cervicitis, approximately 20% of the cases were positive; positivity was higher with CIN1 (25%) and, in women with CIN2 or more advanced lesions, almost 70% of the cytological preparations were positive for the double stain. The results are consistent with other studies in which p16/Ki-67 activity in a colposcopy population had similar associations [24].

A possible relationship between Ct and either cytological or histopathological alterations characteristic of HPV infection remains to be clarified. An association between Ct and a persistent HPV infection may occur preferentially in high-risk women, while a Ct infection may be associated with the clearance of an HPV infection in women who develop an intense inflammatory response to Ct. The identification of women in whom a concurrent Ct infection may be beneficial or detrimental to HPV persistence and progression remains to be clarified [21, 25, 26].

## Conclusion

Ct infection in our study population had a frequency of 15.6% and was associated with age and number of lifetime sexual partners. It was not associated with p16/Ki-67 expression in women with CIN. A possible relationship between Ct and either cytological or histopathological alterations leading to development or persistence of HPV infection remains to be clarified.

## Abbreviations

AGC: Atypical glandular cells
ASCUS: Atypical squamous cells of undetermined significance
CIN: Cervical intraepithelial lesions
CIN 1: Cervical intraepithelial neoplasia 1
CIN2/3: Cervical intraepithelial neoplasia 2 and 3
Ct: *Chlamydia trachomatis*
HPV: Human papillomavirus
HSIL: High-grade squamous intraepithelial lesions
LSIL: Low-grade lesions
SCC: Squamous cell carcinoma
